# Crystal Structure of the Recombination Mediator Protein RecO from *Campylobacter jejuni* and Its Interaction with DNA and a Zinc Ion

**DOI:** 10.3390/ijms23179667

**Published:** 2022-08-26

**Authors:** Su-jin Lee, Han Byeol Oh, Sung-il Yoon

**Affiliations:** 1 Division of Biomedical Convergence, College of Biomedical Science, Kangwon National University, Chuncheon 24341, Korea; 2 Institute of Bioscience and Biotechnology, Kangwon National University, Chuncheon 24341, Korea

**Keywords:** RecO, *Campylobacter jejuni*, recombination, ssDNA, crystal structure

## Abstract

Homologous recombination is involved in repairing DNA damage, contributing to maintaining the integrity and stability of viral and cellular genomes. In bacteria, the recombination mediator proteins RecO and RecR are required to load the RecA recombinase on ssDNA for homologous recombination. To structurally and functionally characterize RecO, we determined the crystal structure of RecO from *Campylobacter jejuni* (cjRecO) at a 1.8 Å resolution and biochemically assessed its capacity to interact with DNA and a metal ion. cjRecO folds into a curved rod-like structure that consists of an N-terminal domain (NTD), C-terminal domain (CTD), and Zn^2+^-binding domain (ZnD). The ZnD at the end of the rod-like structure coordinates three cysteine residues and one histidine residue to accommodate a Zn^2+^ ion. Based on an extensive comparative analysis of RecO structures and sequences, we propose that the Zn^2+^-binding consensus sequence of RecO is CxxC…C/HxxC/H/D. The interaction with Zn^2+^ is indispensable for the protein stability of cjRecO but does not seem to be required for the recombination mediator function. cjRecO also interacts with ssDNA as part of its biological function, potentially using the positively charged patch in the NTD and CTD. However, cjRecO displays a low ssDNA-binding affinity, suggesting that cjRecO requires RecR to efficiently recognize ssDNA for homologous recombination.

## 1. Introduction

Homologous recombination plays a critical role in maintaining genome integrity and stability by repairing DNA damage, including dsDNA breaks and ssDNA gaps, which is caused by radiation and chemical mutagens [1,2,3]. In addition, homologous recombination contributes to genetic diversity and evolution because it can occur between two DNA segments that are similar but not identical in sequence [4]. Homologous recombination is observed in all cellular organisms and even in DNA and RNA viruses. The key step of homologous recombination is mediated by evolutionarily conserved recombinases, such as phage UvsX, bacterial RecA, and eukaryotic Rad51 proteins. Recombinases form a helical nucleoprotein filament with ssDNA and search for homologous sequences, promoting strand exchange for homologous recombination [5,6]. A recombinase is loaded onto ssDNA with the assistance of recombination mediator proteins, including phage UvsY, bacterial RecFOR, and eukaryotic Rad52 proteins.

In *Escherichia coli*, the RecFOR and RecBCD pathways trigger RecA loading on ssDNA and are involved in the repair of ssDNA gaps and dsDNA breaks, respectively [7,8]. The RecFOR proteins have also been shown to facilitate dsDNA break repair when the RecBCD pathway is blocked [9]. For RecFOR-mediated DNA repair, the RecQ helicase unwinds dsDNA, and the RecJ exonuclease degrades the 5′ end of the DNA, extending the ssDNA gap and allowing ssDNA-binding protein (SSB) to interact with ssDNA [10]. RecO and RecR are recruited to SSB-coated ssDNA and load RecA on the ssDNA in place of SSB [11,12,13]. RecF has been shown to bind the ssDNA–dsDNA junction and stimulate the function of RecO and RecR [14,15]. However, some bacterial species, including *Campylobacter jejuni* and *Helicobacter pylori*, lack the *recF* gene and contain only the *recO* and *recR* genes, suggesting that RecF is not a prerequisite for homologous DNA repair [8,16,17].

RecO is a key recombination mediator protein that has DNA- and RecR-binding capacities [18,19,20]. RecO alone promotes DNA annealing, and it stimulates RecA loading on ssDNA when complexed with RecR [21,22,23]. As suggested by the requirement of RecO for recombination, the *recO* gene is ubiquitously found throughout bacteria. However, RecO orthologs differ in size, with 200–270 residues, and are highly diverse in sequence. The sequence identity decreases below 20% between RecO orthologs from different phyla. For example, the RecO proteins of *E. coli* and *Deinococcus radiodurans* (ecRecO and drRecO, respectively) from the Pseudomonadota and Deinococcota phyla, respectively, share only 15.4% sequence identity. Consistently, ecRecO and drRecO differ in their binding to SSB and a Zn^2+^ ion [19,20,24]. ecRecO binds the C-terminal tail of SSB and cannot interact with a Zn^2+^ ion, whereas drRecO is not able to recognize SSB and binds a Zn^2+^ ion [19,20,24]. Moreover, ecRecO and drRecO display different ssDNA-binding affinities [19].

Despite the key role of highly diverse RecO proteins in the initiation of homologous recombination, structural studies have been limited to ecRecO and drRecO [13,18,19,20,24]. The drRecO structure was determined alone as a monomer or in a 2:4 complex with RecR [13,18,19,20]. ecRecO was structurally defined only in complex with the C-terminal tail of SSB [24]. Therefore, the structural and functional diversities of RecO have not been fully explored.

*C. jejuni* belongs to the Campylobacterota phylum along with *H. pylori* and causes food-borne enteritis in humans. *C. jejuni* also expresses RecO protein, which shares a low sequence identity (≤17%) with drRecO and ecRecO. To address the structural and functional diversities of RecO, we determined the crystal structure of *C. jejuni* RecO (cjRecO) and performed a comparative analysis of the RecO structures. In addition, through biophysicochemical analyses, we addressed the unique DNA- and Zn^2+^-binding patterns of cjRecO that are distinct from those of ecRecO and drRecO.

## 2. Results and Discussion

### 2.1. Overall Structure of cjRecO

**Figure 1:**
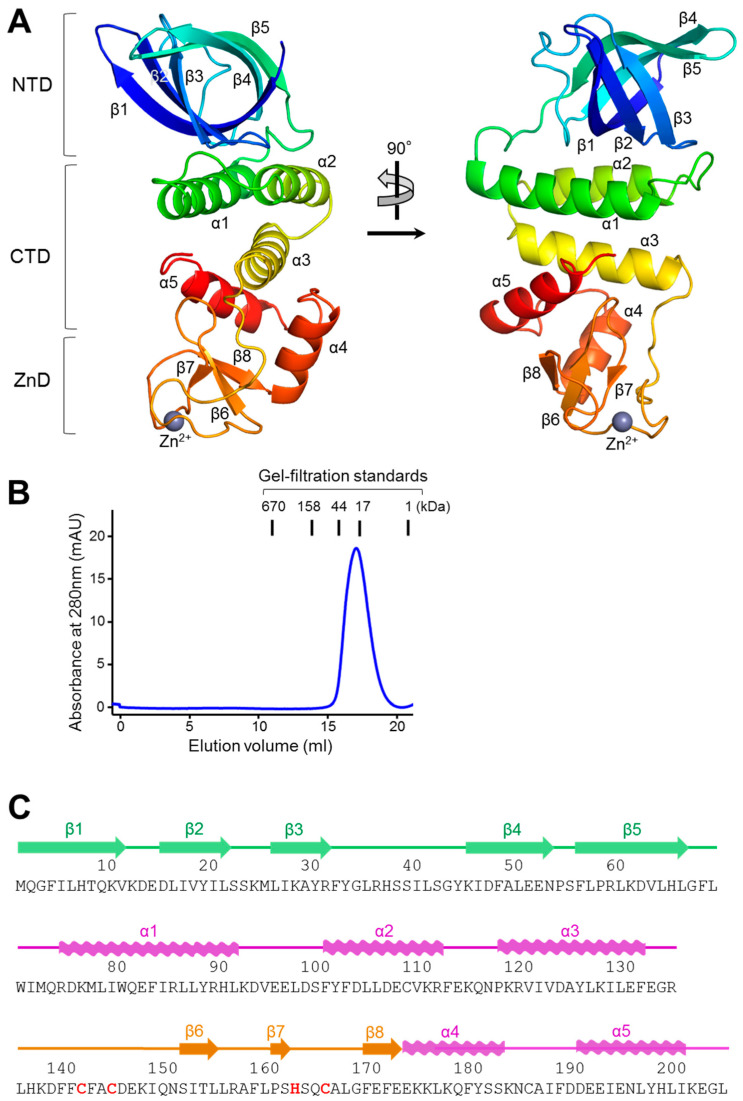
Overall structure and sequence of cjRecO. (**A**) Monomeric structure of cjRecO in rainbow ribbons (N-terminus, blue; C-terminus, red). A Zn^2+^ ion is depicted as a light blue sphere in the ZnD. (**B**) cjRecO monomer identified by gel-filtration chromatography. The data are representative of three independent experiments that yielded similar results. (**C**) Amino acid sequence of cjRecO. The Zn^2+^-coordinating residues of cjRecO are colored red. The secondary structures of cjRecO (α-helices, waves; β-strands, arrows) are shown above the sequence in domain-specific colors (NTD, green; CTD, magenta; ZnD, orange).

The cjRecO protein was recombinantly expressed in *E. coli* cells and purified to homogeneity by chromatographic methods. cjRecO was crystallized in the presence of PEG 3350 in space group *P*2_1_2_1_2_1_. The crystal structure of cjRecO was determined by single-wavelength anomalous diffraction (SAD) phasing and refined to an R_free_ value of 20.8% at a 1.8 Å resolution (Table 1 and Table 2). One cjRecO chain was observed in the asymmetric unit of the cjRecO crystal (Figure 1A). In gel-filtration chromatography, the cjRecO protein was eluted as a monomer (calculated molecular weight, 25.0 kDa) between the 17- and 44-kDa protein standards (Figure 1B). Consistently, ecRecO and drRecO were shown to exist as monomers [11,20].

The cjRecO structure contains all the residues of the cjRecO polypeptide chain (residues 1–204) and takes the shape of a curved rod consisting of five α-helices and eight β-strands (Figure 1A,C). The curved rod-like structure can be divided into three parts: an N-terminal domain (NTD; residues 1–69), a C-terminal domain (CTD; residues 70–134 and 173–204), and a Zn^2+^-binding domain (ZnD; residues 135–172). The CTD is located in the middle of the cjRecO structure between the NTD and ZnD, which form the ends of the cjRecO structure. The NTD is composed of five β-strands (β1–β5), which fold into a closed barrel defined as an oligonucleotide/oligosaccharide-binding (OB) fold [25]. The ZnD contains one three-stranded β-sheet (β6–β8) that is decorated with extended loops, which coordinate a Zn^2+^ ion. In contrast to the NTD and ZnD, the CTD is entirely α-helical with three N-terminal α-helices (α1–α3) and two C-terminal α-helices (α4–α5). The interdomain interfaces of cjRecO are extensive and primarily composed of hydrophobic residues in the center. The hydrophobic interdomain residues combined with the internal residues of each domain form a continuous hydrophobic core that penetrates through the three domains. Thus, each domain of cjRecO would be unstable when left alone without the other domains, and the entire cjRecO polypeptide chain functions as a single entity.

### 2.2. Structural Comparison of RecO Orthologs

**Figure 2:**
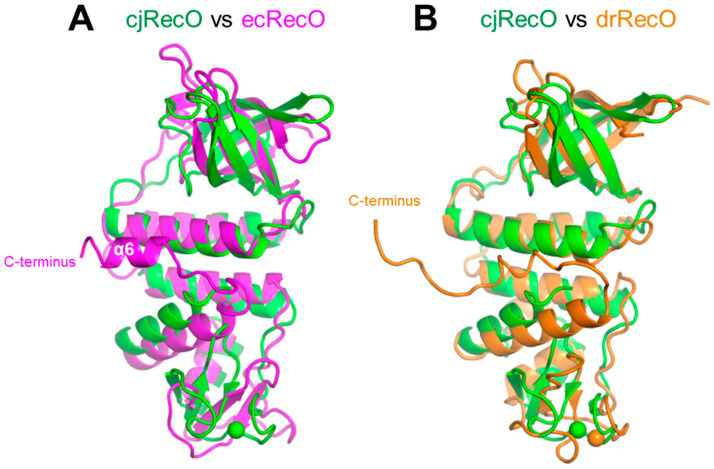
Structural comparison of cjRecO with ecRecO and drRecO. (**A**) Overlay of the cjRecO (green ribbons) and ecRecO (magenta ribbons; PDB ID 3Q8D) structures. A Zn^2+^ ion in the cjRecO structure is depicted as a green sphere. The orientation of cjRecO is identical to that shown in the right side of Figure 1A. (**B**) Overlay of the cjRecO (green ribbons) and drRecO (orange ribbons; PDB ID 1U5K) structures. Zn^2+^ ions in the cjRecO and drRecO structures are depicted as green and orange spheres, respectively. The orientation of cjRecO is identical to that shown in the right side of Figure 1A.

cjRecO from the Campylobacterota phylum exhibits sequence identities of ~17% and ~16% with the structurally defined ecRecO and drRecO from the Pseudomonadota and Deinococcota phyla, respectively. Despite the extremely low sequence identity, the cjRecO structure folds into a curved rod-like three-domain structure as observed for ecRecO and drRecO but with relatively high root-mean-square deviation (RMSD) values (2.7–3.0 Å for 173–185 Cα atoms) (Figure 2) [11,20,24]. Structural differences are observed throughout the three domains. In particular, the ZnD exhibits the highest structural deviation presumably due to its high coil content (~74% coils; ~26% β-strands). The cjRecO ZnD can be overlaid on the drRecO ZnD only for 20 residues (RMSD, ~2.4 Å) from the 38 ZnD residues and is not superimposable on the ecRecO ZnD.

cjRecO is ~40 residues shorter than ecRecO and drRecO and lacks the extended structure that is observed at the C-terminal tail of ecRecO and drRecO (Figure 2 and Figure S1). In the ecRecO structure, the α-helix corresponding to the cjRecO α5 helix is linked to the ecRecO-specific C-terminal helix (α6 in Figure 2A) through a loop, and these two α-helices are employed to bind SSB, contributing to the recruitment of ecRecO to ssDNA for the initiation of recombination (Figure 2A) [24]. Because cjRecO lacks the α6 helix and arranges the α5 helix in a different orientation from that of ecRecO, the C-terminal region of cjRecO does not seem to be involved in the direct interaction with SSB (Figure 2A). The C-terminal region of drRecO also adopts a different conformation from that of ecRecO and was shown not to mediate SSB binding (Figure 2B).

### 2.3. Zn^2+^ Binding by cjRecO

drRecO interacts with a Zn^2+^ ion using four cysteine residues (C153, C156, C173, and C176) in the ZnD [19,20]. Because cjRecO orthologs are highly heterogeneous in sequence, particularly in the ZnD, and exhibit multiple gaps in residue type-based sequence alignment, Zn^2+^ binding by RecO cannot be predicted using sequence information alone (Figure S2). cjRecO contains only three cysteine residues (C141, C144, and C165) in the ZnD. The first two cysteine residues of cjRecO (C141 and C144) are aligned with the Zn^2+^-binding-coordinating cysteine residues of drRecO (C153 and C156), whereas the third cysteine of cjRecO (C165) and its neighboring residues are not well aligned with the drRecO sequence because of low-sequence-identity-mediated gaps. Therefore, the structural information of cjRecO is required to clarify whether cjRecO has Zn^2+^-ion-binding capacity. Moreover, to define the Zn^2+^-binding consensus sequence of RecO proteins, an additional RecO structure in complex with Zn^2+^ is needed.

**Figure 3:**
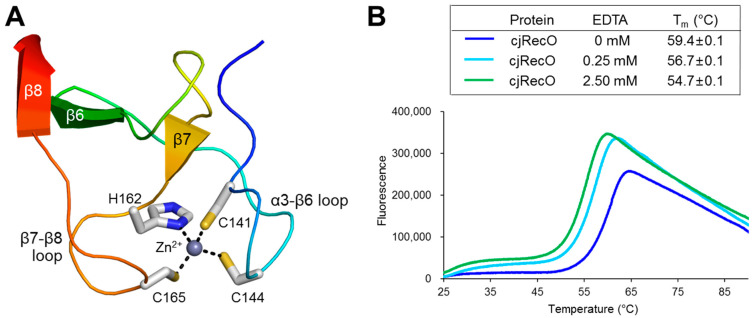
Zn^2+^-binding capacity of cjRecO. (**A**) Zn^2+^ coordination by cjRecO residues. The Zn^2+^ ion and Zn^2+^-coordinating cjRecO residues are represented by a light blue sphere and gray sticks, respectively, in the cjRecO ZnD structure (rainbow ribbons). The orientation of the figure is similar to that shown in the right side of Figure 1A. (**B**) Thermal shift assay of cjRecO in the absence and presence of the divalent ion-chelating reagent EDTA. The T_m_ value of the cjRecO protein decreased in the presence of EDTA, suggesting that Zn^2+^ binding enhances the protein stability of cjRecO. The data in the figure are representative of three independent experiments that yielded similar results. T_m_ values are shown as means ± S.D. from three independent experiments.

**Figure 4:**
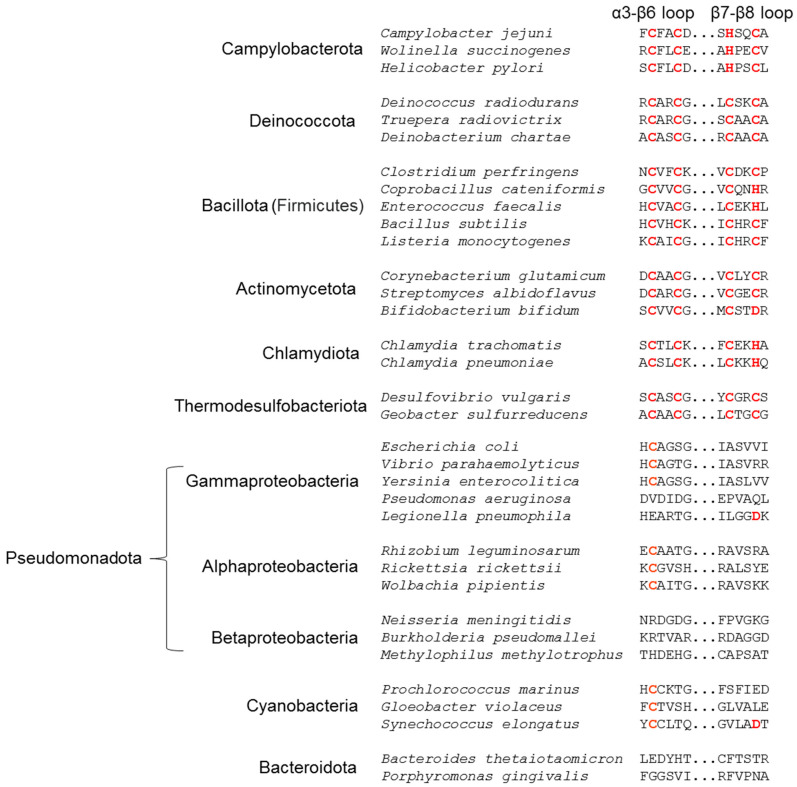
The sequence of RecO proteins from diverse taxa at the Zn^2+^-binding motif represented by the CxxC…C/HxxC/H/D sequence. The conserved residues at the Zn^2+^-binding motif and their equivalent identical sequences are colored red.

In the crystal structure of cjRecO, a high electron density peak that corresponds to a Zn^2+^ ion was identified. The Zn^2+^ ion is located at one end of the rod-like cjRecO structure in the coil-rich ZnD and coordinated by two cysteine residues (C141 and C144) from the α3-β6 loop and by one histidine residue (H162) and one cysteine residue (C165) from the β7-β8 loop (Figure 1A and Figure 3A). Thus, cjRecO uses the CxxC…HxxC motif to coordinate a Zn^2+^ ion in a pattern similar to the Zn^2+^-binding CxxC…CxxC motif of drRecO (Figure 1C, Figure 3A, and Figure S1). The inspection of RecO sequences from diverse taxa, combined with the structural information, allowed us to define the consensus sequence for Zn^2+^ binding and to predict the Zn^2+^-binding ability of each RecO protein. RecO proteins from the Campylobacterota, Deinococcota, Bacillota, Actinomycetota, Chlamydiota, and Thermodesulfobacteriota phyla are highly likely to interact with Zn^2+^, using the CxxC…C/HxxC/H/D consensus sequence (Figure 4). However, RecO proteins from the Pseudomonadota, Cyanobacteria, and Bacteroidota phyla lack the Zn^2+^-binding consensus sequence and would not interact with a Zn^2+^ ion (Figure 4). Indeed, *E. coli*, which belongs to the Pseudomonadota phylum, lacks the consensus sequence and was shown not to bind a Zn^2+^ ion. Therefore, the Zn^2+^ interaction does not seem to be directly involved in the common biological function of RecO as a recombination mediator and may play other roles, for example, in protein folding.

In the cjRecO structure, the Zn^2+^ ion tethers the two extended loops of the coil-rich ZnD and thus seems to stabilize the hydrophobic core of cjRecO at one end of the rod-like cjRecO structure (Figure 3A). To determine the structural function of Zn^2+^ in cjRecO, the protein stability of cjRecO was analyzed by a thermal shift assay in the absence and presence of a divalent cation-chelating agent EDTA (Figure 3B). In the absence of EDTA, cjRecO displayed a canonical one-step denaturation curve, and its thermal shift was observed between 55 and 65 °C with a melting temperature (T_m_; temperature at 50% denaturation) of 59.4 °C. However, when EDTA was added to abstract a Zn^2+^ ion from cjRecO, cjRecO displayed a two-step denaturation curve. The first transition was observed between 25 and 30 °C with a gentle fluorescence increase, presumably due to the disruption of the small Zn^2+^-binding fold in the ZnD. The second transition was steep between 50 and 60 °C and seems to be generated by global disintegration of the entire hydrophobic core of cjRecO. The EDTA-mediated abstraction of a Zn^2+^ ion from cjRecO decreased the T_m_ value of cjRecO by 2.7 °C at 0.25 mM EDTA and by 4.7 °C at 2.5 mM EDTA. In contrast to cjRecO, the control protein *Xanthomonas campestris* FliD (xcFliD), which is not able to interact with a divalent metal ion, displayed similar thermal shift patterns irrespective of the EDTA concentration (Figure S3). These observations indicate that Zn^2+^ binding is required to maintain the protein stability of cjRecO.

### 2.4. ssDNA Binding by cjRecO

**Figure 5:**
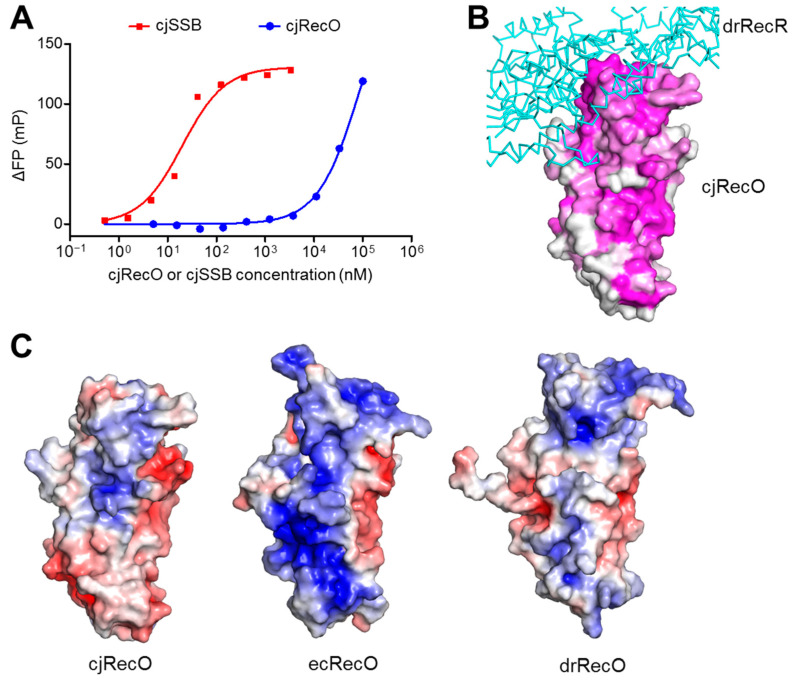
ssDNA binding by cjRecO. (**A**) Interaction of cjRecO and cjSSB with ssDNA identified by the FP assay. The data are representative of three independent experiments that yielded similar results. (**B**) Sequence conservation of RecO proteins on the cjRecO structure. The cjRecO structure is shown as surfaces that represent the sequence conservation of cjRecO orthologs (high sequence conservation, magenta; low sequence conservation, white). To show the putative RecR-binding regions of cjRecO, the drRecR structure from the drRecO-drRecR complex (PDB ID 4JCV) is shown as cyan Cα traces with the cjRecO structure. The orientation of cjRecO is identical to that shown in the right side of Figure 1A. The backside view of the figure is shown in Figure S4. (**C**) Electrostatic potential surfaces (positive, blue; neutral, white; negative, red) of cjRecO (left panel), ecRecO (middle panel; PDB ID 3Q8D), and drRecO (right panel; PDB ID 1U5K).

drRecO and ecRecO have been shown to recognize ssDNA [20,22,24]. To examine whether cjRecO also interacts with ssDNA, a fluorescence polarization (FP) assay was performed for cjRecO protein using fluorescein-labeled ssDNA. In the FP assay, cjRecO protein began to exhibit detectable ssDNA binding at a concentration of ~1 μM and increased its ssDNA-binding level at higher concentrations (Figure 5A). Although a complete saturation curve was not obtained up to 100 μM cjRecO, the dissociation equilibrium constant (K_d_) value for the cjRecO–ssDNA interaction is expected to be at least 20 μM. In contrast, the *C. jejuni* SSB protein (cjSSB) exhibited a K_d_ value of 21.4 ± 2.5 nM, with at least a 1000-fold higher ssDNA-binding affinity than that of cjRecO. These observations indicate that cjRecO interacts with ssDNA with an extremely low ssDNA-binding affinity.

To identify the ssDNA-binding site of RecO, we analyzed the residue conservation in RecO orthologs and calculated the electrostatic potentials on the RecO structures. RecO proteins exhibit high sequence conservation on one side of the rod-like structure (Figure 5B and Figure S4). Notably, in the ecRecO structure, these conserved regions display high positive electrostatic surface potentials throughout the three domains, although a portion of them is presumably used to interact with RecR (Figure 5C, middle). These observations suggest that ecRecO employs the positively charged, extended patch to interact with negatively charged ssDNA. The cjRecO and drRecO structures also exhibit positive electrostatic potentials on the same side in the NTD and CTD (Figure 5C). Consistently, the NTD contains the OB fold, which has been shown to interact with oligonucleotides or oligosaccharides. However, the cjRecO and drRecO structures are characterized by lower positive charges than ecRecO. In particular, in the cjRecO structure, the positive patch is limited to NTD and CTD and does not extend to ZnD (Figure 5C, left). These structural findings are consistent with the extremely low ssDNA-binding affinity of cjRecO and the lower ssDNA-binding affinity of drRecO than that of ecRecO [19]. The low ssDNA-binding affinity of cjRecO implies that RecR plays a more critical role in ssDNA recognition by the RecO-RecR complex in *C. jejuni*. Taken together, cjRecO recognizes ssDNA potentially using the conserved, elongated patch in the NTD and CTD but with lower affinity than that of ecRecO.

In conclusion, cjRecO shares a low sequence identity with its orthologs from other phyla and exhibits various structural differences despite overall similar three-domain structures. Moreover, cjRecO is distinct from drRecO and ecRecO in the binding of a metal ion and ssDNA. Thus, for recombination, cjRecO is highly likely to use a unique molecular mechanism that is not observed in drRecO and ecRecO. Because RecO functions in complex with RecR, the RecO–RecR interaction needs to be structurally and biophysically addressed to reveal the unique recombination-promoting mechanism of *C. jejuni* RecOR.

## 3. Materials and Methods

### 3.1. Construction of the Protein Expression Plasmid

To construct the cjRecO protein expression plasmid, the cjRecO-encoding gene was amplified by PCR from the genomic DNA of *C. jejuni* subsp. *jejuni* (ATCC 33291) using Pfu DNA polymerase (Enzynomics) and DNA primers containing a recognition site of the *Bam*HI or *Sal*I restriction enzyme (forward primer, 5′-TAAGGATCCGATGCAAGGCTTTATACTTCATACTCAAAAAG-3′); reverse primer, 5′-GCCGATGTCGACTCATAAACCTTCCTTAATCAAATGATATAAATTTTC-3′) with 30 cycles of denaturation (95 °C, 30 s), annealing (55° C, 45 s), and extension (72 °C, 150 s). The PCR product was then digested using the BamHI and SalI restriction enzymes. The resulting DNA fragment was ligated using T4 DNA ligase into the pET49b plasmid, which was modified to express recombinant protein in fusion with a hexahistidine tag and a thrombin cleavage site at the N-terminus [26]. The ligation product was transformed into *E. coli* DH5α cells. A transformant was selected in the presence of kanamycin, and the nucleotide sequence of the insert in the cjRecO protein expression plasmid was confirmed through DNA sequencing. The cjSSB protein expression plasmid was also generated in a similar manner to that of the cjRecO protein expression plasmid, except that cjSSB was designed to be expressed in fusion with a hexahistidine tag and a TEV protease cleavage site at the N-terminus.

### 3.2. Protein Expression and Purification

To obtain recombinant cjRecO protein, the cjRecO expression plasmid was transformed into *E. coli* BL21 (DE3) cells. The *E. coli* cells containing the cjRecO expression plasmid were grown at 37 °C in LB medium supplemented with kanamycin. When the absorbance of the culture at 600 nm reached 0.6, isopropyl β-D-1-thiogalactopyranoside was added to the culture at a final concentration of 1 mM to induce cjRecO protein overexpression. Cells were further cultured at 18 °C for 18 h. The resulting cells containing cjRecO protein were harvested by centrifugation and lysed by sonication in 50 mM Tris, pH 8.0, 200 mM NaCl, and 5 mM β-mercaptoethanol (βME). The hexahistidine-tagged cjRecO protein was first purified from the cell lysate by affinity chromatography using Ni-NTA resin. cjRecO protein was eluted from Ni-NTA resin in a stepwise manner using a solution with 50–300 mM imidazole, 50 mM Tris, pH 8.0, 200 mM NaCl, and 5 mM βME. The eluted cjRecO protein was dialyzed against 20 mM Tris, pH 8.0, and 5 mM βME. The dialyzed cjRecO protein was treated with thrombin to remove the hexahistidine tag. After thrombin cleavage, the tag-free cjRecO protein was purified via anion exchange chromatography using a Mono Q 10/100 column (GE Healthcare, Chicago, IL, USA) with an NaCl gradient (0–500 mM) in 20 mM Tris, pH 8.0, and 5 mM βME. The purified cjRecO protein was concentrated using a centrifugal filter for crystallization.

To obtain the selenomethionine (SeMet)-incorporated cjRecO protein (SeMet-cjRecO), the cjRecO expression plasmid was transformed into the *E. coli* strain B834 (DE3). The SeMet-cjRecO protein was overexpressed in *E. coli* B834 (DE3) cells using nutrient-supplemented M9 minimal medium containing 40 μg/mL L-SeMet. The expressed SeMet-cjRecO protein was purified using Ni-NTA affinity chromatography and anion exchange chromatography in an identical manner to that of the native cjRecO protein.

cjSSB protein was expressed and purified as described for the native cjRecO protein except the protease digestion step. After dialysis, the hexahistidine-tagged cjSSB protein was digested with TEV protease, and the cleaved cjSSB protein was separated from the hexahistidine tag and the undigested cjSSB protein via Ni-NTA affinity chromatography. The tag-free cjSSB protein was further purified by anion exchange chromatography in an identical manner to that of the cjRecO protein.

### 3.3. Protein Crystallization and X-ray Diffraction

cjRecO crystallization was performed at 18 °C via a sitting-drop vapor-diffusion method by equilibrating an equivolume mixture of protein and a crystallization solution against a reservoir solution in a Cryschem plate (Hampton Research, Aliso Viejo, CA, USA). The native cjRecO protein was crystallized in a solution containing 20% PEG 3350 and 0.2 M sodium formate, and the resulting crystal was cryoprotected in 22% PEG 3350, 0.2 M sodium formate, and 25% ethylene glycol. SeMet-cjRecO crystals were obtained using 22% PEG 3350 and 0.1 M Tris, pH 8.5, and were subjected to cryoprotection in 25% PEG 3350, 0.1 M Tris, pH 8.5, and 25% glycerol. The cryoprotected crystal was flash-cooled under a nitrogen gas cryostream. The X-ray diffraction of the cjRecO crystal was carried out at the Pohang Accelerator Laboratory, beamline 7A. The diffraction data were processed using the HKL2000 program [27].

### 3.4. Structure Determination and Analysis

The SeMet-cjRecO structure was determined by SAD phasing using the AutoSol program in the Phenix package (Table 1) [28]. The partial structure of SeMet-cjRecO was used as a search model to determine the structure of native cjRecO by molecular replacement with the Phaser program [29]. The final structure of native cjRecO protein in complex with a Zn^2+^ ion was obtained through iterative cycles of model building and refinement using the Coot and Phenix.refine programs, respectively (Table 2) [30,31]. The presence of the Zn^2+^ ion in the cjRecO structure was confirmed by an X-ray fluorescence scattering scan for a cjRecO crystal. The Zn^2+^ ion in the cjRecO structure exhibited a high electron density peak (16.8σ) in the 2Fo-Fc map.

### 3.5. Gel-Filtration Chromatography

To analyze the oligomeric state of cjRecO, gel-filtration chromatography was performed in a running solution with 20 mM Tris, pH 8.0, 150 mM NaCl, and 5 mM βME. The purified cjRecO protein (100 μg in 300 μL) was loaded onto a Superdex 200 10/300 column. Protein elution was monitored by measuring the UV absorbance at 280 nm. For size estimation, gel-filtration standards (Bio-Rad, Hercules, CA, USA) were independently loaded onto the column.

### 3.6. Thermal Shift Assay

To investigate whether Zn^2+^ is essential for the protein stability of cjRecO, the T_m_ value of cjRecO was determined in the presence or absence of EDTA by a thermal shift assay. Protein was incubated with EDTA (0, 0.25, or 2.5 mM) at 18 °C for 30 min and then mixed with SYPRO Orange. Fluorescence (excitation wavelength, 470 ± 15 nm; emission wavelength, 520 ± 15 nm) was measured at 25–99 °C using a QuantStudio 1 Real-Time PCR System. The T_m_ value was derived with the wTSA-CRAFT software [32]. The xcFliD protein was used as a control protein.

### 3.7. FP Assay

An FP assay was performed to determine the ssDNA-binding affinities of cjRecO and cjSSB. A fluorescein-labeled 40-mer ssDNA (5′-TTATAGGCATATAGGAGTAATTTTCTTGGGCTATGCAGTA-3′; 0.8 nM) was incubated with each protein at various concentrations in 20 mM Tris, pH 8.0, 30 mM NaCl, and 5 mM βME, and then the FP of the fluorescein-labeled ssDNA was measured using an Infinite F200 PRO instrument (Tecan, Männedorf, Switzerland). A K_d_ value was derived with the Prism software (version 5.01, GraphPad, San Diego, CA, USA) using a one-site binding model.

## Figures and Tables

**Table 1:** Crystallographic statistics for SAD phasing of the SeMet-cjRecO structure.

	SeMet-cjRecO
**Data Collection**	
Space group	*P*2_1_2_1_2_1_
Cell parameters	
a (Å)	39.17
b (Å)	61.88
c (Å)	97.53
Wavelength (Å)	0.9792
Resolution (Å)	30.00–1.90
Highest resolution (Å)	1.93–1.90
No. unique reflections	19,561 (957) ^a^
R_merge_ (%) ^b^	11.8 (96.0) ^a^
R_meas_ (%) ^c^	12.3 (99.8) ^a^
R_pim_ (%) ^d^	3.4 (26.8) ^a^
CC_1/2_ ^e^	0.996 (0.908) ^a^
I/sigma(I)	49.8 (5.9) ^a^
Completeness (%)	99.9 (100.0) ^a^
Redundancy	13.4 (13.6) ^a^

^a^ Numbers in parentheses were calculated from data for the highest resolution shell. ^b^ R_merge_ = Σ_hkl_Σ_i_ |I_i_(hkl) − <I(hkl)>|/Σ_hkl_Σ_i_ I_i_(hkl); ^c^ R_meas_ = Σ_hkl_ {N(hkl)/[N(hkl) − 1]}^1/2^ Σ_i_ |I_i_(hkl) − <I(hkl)>|/Σ_hkl_Σ_i_ I_i_(hkl); ^d^ R_pim_ = Σ_hkl_ {1/[N(hkl) − 1]}^1/2^ Σ_i_ |I_i_(hkl) − <I(hkl)>|/Σ_hkl_Σ_i_ I_i_(hkl); ^e^ Correlation coefficient between intensities from random half-data sets.

**Table 2:** Crystallographic statistics of the native cjRecO structure.

	Native cjRecO
**Data collection**	
Space group	*P*2_1_2_1_2_1_
Cell parameters	
a (Å)	39.15
b (Å)	61.71
c (Å)	97.20
Wavelength (Å)	0.9792
Resolution (Å)	30.00–1.80
Highest resolution (Å)	1.83–1.80
No. unique reflections	22,418 (1110) ^a^
R_merge_ (%) ^b^	8.0 (58.4) ^a^
R_meas_ (%) ^c^	8.8 (64.5) ^a^
R_pim_ (%) ^d^	3.7 (26.9) ^a^
CC_1/2_ ^e^	0.995 (0.838) ^a^
I/sigma(I)	33.1 (3.9) ^a^
Completeness (%)	99.6 (99.7) ^a^
Redundancy	5.7 (5.6) ^a^
**Refinement**	
Resolution (Å)	30.00–1.80
No. of reflections (work)	21,294
No. of reflections (test)	1080
R_work_ (%) ^f^	17.9
R_free_ (%) ^g^	20.8
No. atoms	
Protein	1734
Zinc	1
Water	109
Average B-value (Å^2^)	29.7
RMSD bonds (Å)	0.006
RMSD angles (°)	0.775
Ramachandran ^h^ (favored)	98.0%
Ramachandran ^h^ (outliers)	0.0%

^a^ Numbers in parentheses were calculated from data for the highest resolution shell. ^b^ R_merge_ = Σ_hkl_Σ_i_ |I_i_(hkl) − <I(hkl)>|/Σ_hkl_Σ_i_ I_i_(hkl); ^c^ R_meas_ = Σ_hkl_ {N(hkl)/[N(hkl) − 1]}^1/2^ Σ_i_ |I_i_(hkl) − <I(hkl)>|/Σ_hkl_Σ_i_ I_i_(hkl); ^d^ R_pim_ = Σ_hkl_{1/[N(hkl) − 1]}^1/2^ Σ_i_ |I_i_(hkl) − <I(hkl)>|/Σ_hkl_Σ_i_ I_i_(hkl); ^e^ Correlation coefficient between intensities from random half-data sets. ^f^ R_work_ = Σ ||F_obs_| − |F_calc_||/Σ |F_obs_| where F_calc_ and F_obs_ are the calculated and observed structure factor amplitudes, respectively. ^g^ R_free_ = as described for R_work_, except that 5% of the total reflections were selected at random and omitted from refinement. ^h^ Calculated using MolProbity (molprobity.biochem.duke.edu, accessed on 29 July 2022).

## Data Availability

The atomic coordinates and the structure factors for RecO (PDB ID 7YMO) have been deposited in the Protein Data Bank (www.rcsb.org, accessed on 29 July 2022).

## Supplementary Materials

The following supporting information can be downloaded at: https://www.mdpi.com/article/10.3390/ijms23179667/s1. References [33,34] are cited in Supplementary Materials.

## Abbreviations

cjRecO: *Campylobacter jejuni* RecO; cjSSB, *C. jejuni* SSB protein; CTD, C-terminal domain; drRecO, *Deinococcus radiodurans* RecO; ecRecO, *Escherichia coli* RecO; FP, fluorescence polarization; K_d_, dissociation equilibrium constant; βME, β-mercaptoethanol; NTD, N-terminal domain; RMSD, root-mean-square deviation; SAD, single-wavelength anomalous diffraction; SeMet, selenomethionine; SeMet-cjRecO, selenomethionine-incorporated cjRecO; SSB, ssDNA-binding protein; T_m_, melting temperature; xcFliD, *Xanthomonas campestris* FliD; ZnD, Zn^2+^-binding domain.
