# Supplementary material for: Crystal Structure of the Recombination Mediator Protein RecO from Campylobacter jejuni and Its Interaction with DNA and a Zinc Ion
Source: Int J Mol Sci. 2022 Aug 26;23(17):9667. doi: 10.3390/ijms23179667 (PMC9456098; doi:10.3390/ijms23179667)
Supplement: Supplementary file 1 [file ijms-23-09667-s001.zip › ijms-1878039-supplementary.pdf]

Figure S1

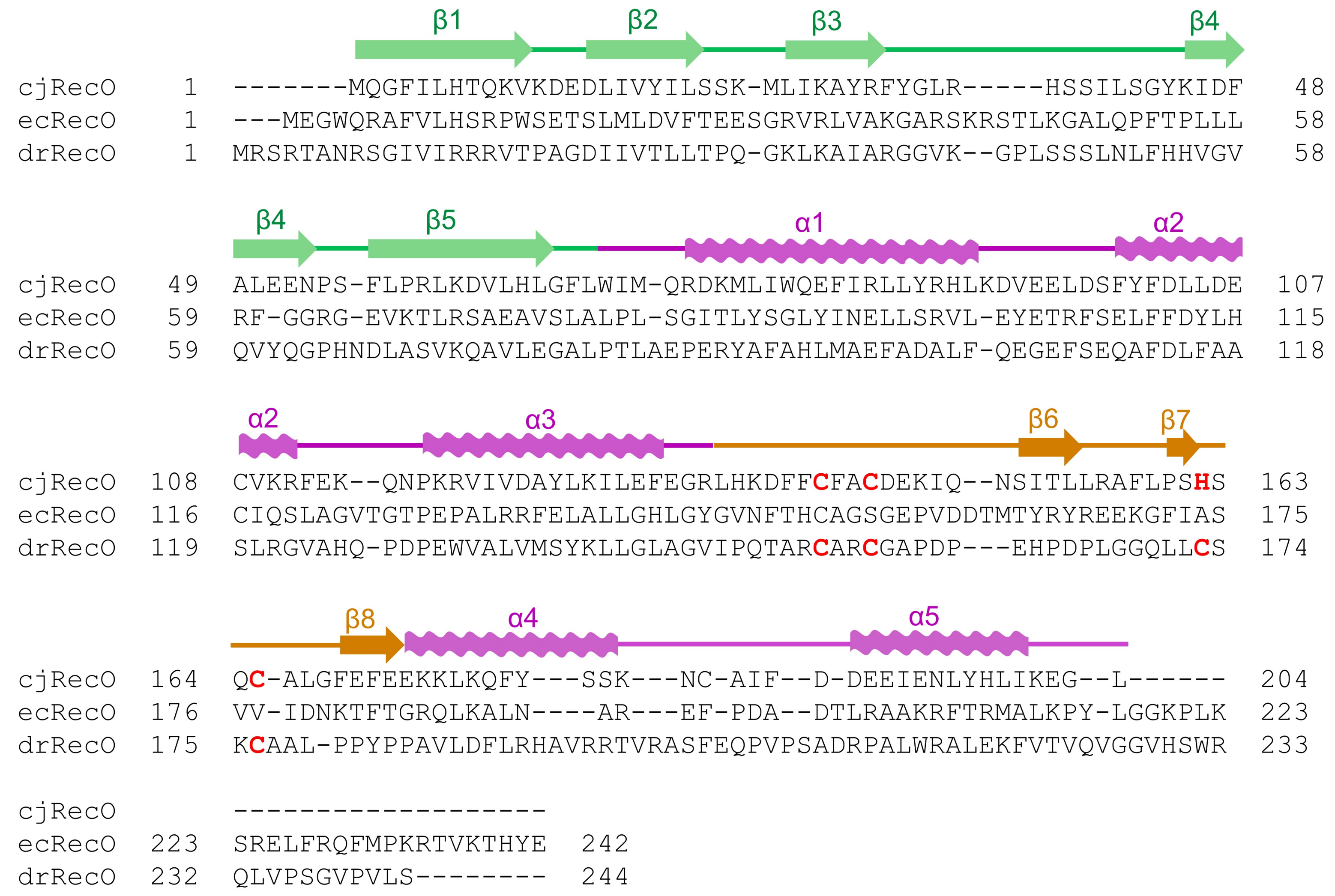

Figure S1. Structure-based sequence alignment of cjRecO, ecRecO, and drRecO. The Zn^{2+}-coordinating residues of cjRecO and drRecO are colored red. The secondary structures of cjRecO (α-helices, waves; β-strands, arrows) are shown above the cjRecO sequence in domain-specific colors (NTD, green; CTD, magenta; ZnD, orange). Structure-based sequence alignment was performed using the PROMALS3D program [33].

Figure S2

```
cjRecO      ---------MQGFILHTQKVKDEDLIVYILSSKMLIKAYRFYGLRHSSILSGYKIDFALE    51
hpRecO      ---------MQGFLLQTQSIRDEDLIVHILTKNQLKTLYRFYGKRHSVLNVGRKIDFEEE    51
rlRecO      -----MQWQDHAIILGVKRHGETSVIAEVMTRDRGRHLGLVRSGR-SR------------    42
bsRecO      -----MLTKCEGIVLRTNDYGETNKIVTLLTREHGKIGVMARGAK-KP------------    42
bpRecO      -------------MLHSYPYRETSLVIDVLTRDHGRIALVAKGAK-RP------------    34
ecRecO      -----MEGWQRAFVLHSRPWSETSLMLDVFTEESGRVRLVAKGAR-SK------------    42
vpRecO      MSNLSAEGFQRCFVLHRRPYSESSLILDVFSEEYGRLTLMAKGAR-SK------------    47
drRecO      -MRSRTANRSG-IVIRRRVTPAGDIIVTLLTPQGK-LKAIARGGVKGP------------    45
bbRecO      ----MTQGKDRFIILKKIKYAEADLIIHGLSPRGEKLAFIARGALKSK------------    44

cjRecO      ENPSFLPRLKDVLHLGFLWIMQRDKM--L-----------------------------IW    80
hpRecO      NDDKFLPKLRNILHLGYIWEREMERL--F-----------------------------FW    80
rlRecO      ---AMQPVLQAGNAVEVIWRARLD-EHLGEFRVEPVTLRAARLMETA--------TAVYG    90
bsRecO      -NSRLSAVSQPFLYGSFLMQKTSG---LG-------TLQQGEMILSMRGIREDLFLTAYA    91
bpRecO      -HSALRGVLQTFQPLSLSWSGKSE---LR-------TLTGAEWVGGMLPLAGDALL--CG    81
ecRecO      -RSTLKGALQPFTPLLLRFGGRGE---VK-------TLRSAEAVSLALPLSGITLY--SG    89
vpRecO      -RSNLKGALQPFTPLLLKWSGKGS---MK-------TLRQAEPISLGLPLFGINLY--SA    94
drRecO      -LSSSLNL---FHHVGVQVY-QGPHNDLA-------SVKQAVLEGALPTLAEP-ERYAFA    92
bbRecO      -KRFGGGVLEPTHFVTFTYKQANEHGQLN-------VLQEATIVNDFPGLRTDYDRLETA    96

cjRecO      QEFIRLLYRHLKDVEELDSFYFDLLDECVKRFEK--QNPKRVIVDAYLKILEFEGRLHKD    138
hpRecO      QRFCALLFRHLEGVHSLDSIYFDTLDDGASKLSK--QHPLRVVLEMYATLLNFEGRLQSY    138
rlRecO      VQAMGALLRLLPERDPH-PHLFDALEVILDHLHN-PADAGELFVRFELAVLNDLGFGLDL    148
bsRecO      AYVAELVDRGTEEKKPN-PYLFEFILESLKQLNE-GTDPDVITFIVQMKMLGVMGLYPEL    149
bpRecO      FYANELLVKFCAREDPH-PPLFQHYLVTLTRLAH-GEPPVQVLRSFERVLLRETGYAMTL    139
ecRecO      LYINELLSRVLEYETRF-SELFFDYLHCIQSLAGVTGTPEPALRRFELALLGHLGYGVNF    148
vpRecO      MYVNELVGRVLMAEVPM-PALFHDYLHALTELAQ-CENPEPALRRFELALLSSMGYGVDF    152
drRecO      HLMAEFADALFQEGE-FSEQAFDLFAASLRGVAH-QPDPEWVALVMSYKLLGLAGVIPQT    150
bbRecO      LHILECVSKVSQEGDRDSEFLFNLLGNTLKVLES-TPDILVLKMHFYLKFLLQQGVVNAE    155

cjRecO      FFCFACDEKIQNSIT-----LLRAFLPSHS--------QCALGFEFEEKKLKQFYS--SK    183
hpRecO      HSCFLCDKKLENSVT-----LAQGFILAHP--------SCLKTKSLDLEKIQAFFR--TQ    183
rlRecO      AECAATGTRSD--LV--YVSPKSGRAVSRAA-GAPWADKMLLLPPFLS----------IE    193
bsRecO      NHCVHCKSQ---DGTFHFSVRDNGFICHRCFEKDPYRIP-IKPQTARLLRLFYYFDLSRL    205
bpRecO      KRTVA-RRAVEPDKLYVFD-PQHGVRDAGGDA--PSHWPVIAGQTLLDMEEDDY------    189
ecRecO      THCAGSGEPVDDTMTYRYR-EEKGFIASVV-----IDNKTFTGRQLKALNAREF------    196
vpRecO      LHCAGTGEPVAPEMTYRYR-EQKGFIASVR-----RDNLTFLGNELIAISERRF------    200
drRecO      ARCARCGAPDPEH----PDPLGGQLLCSKCAALPPYPPAVLD---------FLRHAVRRT    197
bbRecO      PWMAPF---------------LKTNL-ADSANLISYRQTVDD---------EMYN-VEQM    189

cjRecO      NCAIFDDEEIEN----LYHLIKEGL-----------------------------------    204
hpRecO      STIDLETEEVEE----LWRTLNLGF-----------------------------------    204
rlRecO      GNHAADFDSLSAAFRLTGFFLHRHVYEP-----------RGIESVAARDGFVQAALKALN    242
bsRecO      GNVSLKEETKAELKQVIDLYYEEY----------------SGLYLKSKRFLDQME-----    244
bpRecO      ----HRAQTVAQSKTLMRFLLNTYL---------------GGTPLATRQILIDLQ-----    225
ecRecO      ----PDADTLRAAKRFTRMALKPYL---------------GGKPLKSRELFRQFM-----    232
vpRecO      ----VTKEQLKAAKRFTRIALKPYL---------------GGKPLKSRELFIQTP-----    236
drRecO      VRASFEQPVPSADRPALWRALEKFVTVQVGGVHSWRQLVPSGVPVLS-------------    244
bbRecO      VKHYLAN----A---TL-------------------------------------------    199

cjRecO      --------------    204
hpRecO      --------------    204
rlRecO      PTPRTLSGPNGVSA    256
bsRecO      -SMKHLMGENKS--    255
bpRecO      -NL-----------    227
ecRecO      -PKRTVKTHYE---    242
vpRecO      -RARSNGK------    243
drRecO      --------------    244
bbRecO      --------------    199
```

Figure S2. Residue type-based sequence alignment of cjRecO, *H. pylori* RecO (hpRecO), *Rhizobium leguminosarum* RecO (rlRecO), *Bacillus subtilis* RecO (bsRecO), *Burkholderia pseudomallei* RecO (bpRecO), ecRecO, *Vibrio parahaemolyticus* RecO (vpRecO), drRecO, and *Bdellovibrio bacteriovorus* RecO (bbRecO). The Zn^{2+}-coordinating residues of cjRecO and drRecO are colored red. The residues of RecO orthologs that are identical to those of cjRecO are highlighted by a cyan background. Residue type-based sequence alignment was performed using the Clustal Omega program [34].

Figure S3

	Protein	EDTA	T_m (°C)
	xcFliD	0 mM	48.2±0.0
	xcFliD	0.25 mM	48.1±0.0
	xcFliD	2.5 mM	48.4±0.1

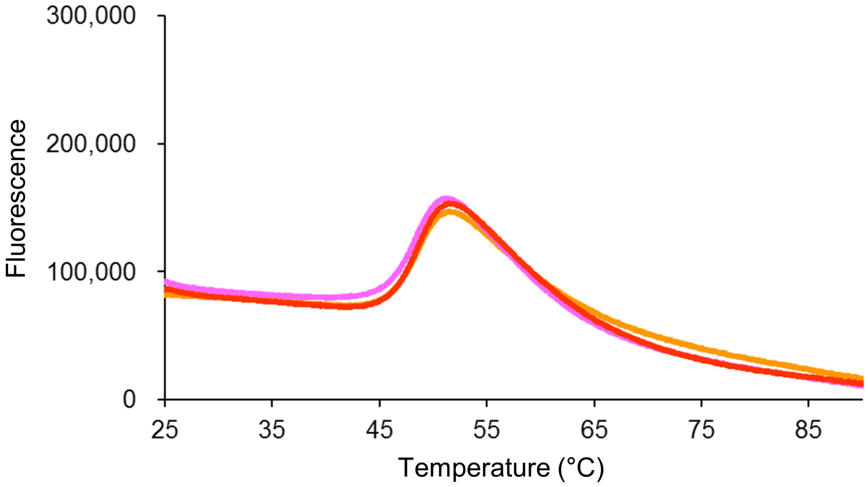

Figure S3. Thermal shift assay of the control protein xcFliD in the absence and presence of EDTA. EDTA did not change the protein stability of xcFliD, which does not have a metal-binding capacity. The data in the figure are representative of three independent experiments that yielded similar results. T_m values are shown as means ± S.D. from the three independent experiments.

Figure S4

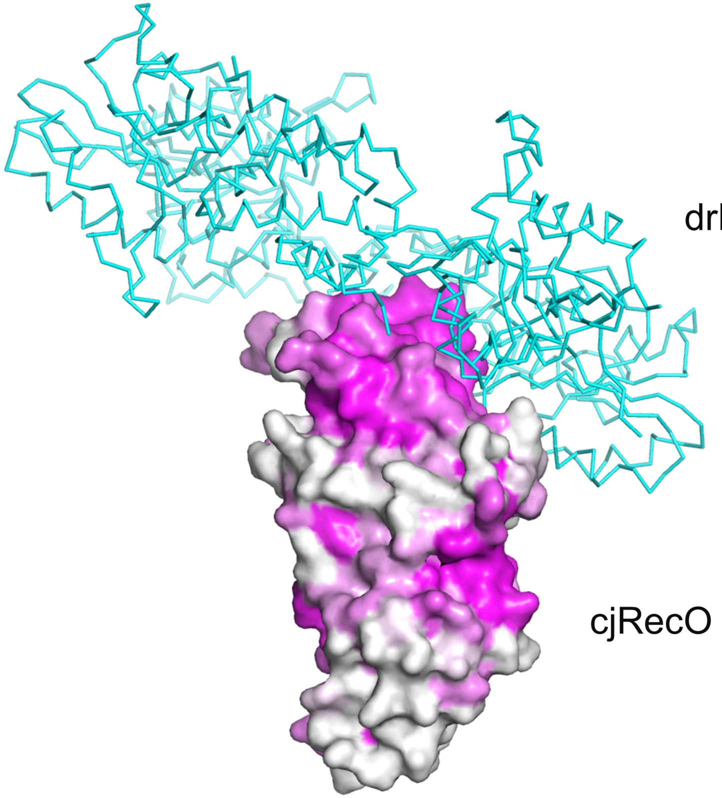

Figure S4. Sequence conservation of RecO proteins on the cjRecO structure in the backside view of Figure 5B. The cjRecO structure is shown as surfaces that represent the sequence conservation of cjRecO orthologs (high sequence conservation, magenta; low sequence conservation, white). The drRecR structure from the drRecO-drRecR complex (PDB ID 4JCV) is depicted as cyan Cα traces with the cjRecO structure. The RecR-binding region of RecO exhibits high sequence conservation.
